# Neuroinflammation and Post-Stroke Depression: Focus on the Microglia and Astrocytes

**DOI:** 10.14336/AD.2024.0214-1

**Published:** 2024-02-14

**Authors:** Weizhuo Lu, Jiyue Wen

**Affiliations:** ^1^Department of Pharmacology, School of Basic Medical Sciences, Anhui Medical University, Hefei, China; ^2^Medical Branch, Hefei Technology College, Hefei, China

**Keywords:** ischemic stroke, PSD, microglia, astrocyte, neuroinflammation

## Abstract

Post-stroke depression (PSD), a frequent and disabling complication of stroke, has a strong impact on almost thirty percent of stroke survivors. The pathogenesis of PSD is not completely clear so far. Neuroinflammation following stroke is one of underlying mechanisms that involves in the pathophysiology of PSD and plays an important function in the development of depression and is regarded as a sign of depression. During the neuroinflammation after ischemic stroke onset, both astrocytes and microglia undergo a series of morphological and functional changes and play pro-inflammatory or anti-inflammatory effect in the pathological process of stroke. Importantly, astrocytes and microglia exert dual roles in the pathological process of PSD due to the phenotypic transformation. We summarize the latest evidence of neuroinflammation involving in PSD in this review, focus on the phenotypic transformation of microglia and astrocytes following ischemic stroke and reveal the dual roles of both microglia and astrocytes in the PSD via modulating the neuroinflammation.

## 1. Introduction

As a medical emergency, ischemic stroke frequently leads to severe neurological dysfunctions and other sequelae. PSD is a prevalent one among all neurological sequelae for it is experienced by approximate one-third of stroke patients [1, 2]. The reduced quality and expectancy of a patient's life with PSD are related to multiple factors, containing cognitive decline, increased fall risk, increased suicide rate, poor response to rehabilitation and dysfunction [3]. In addition to the decreased quality of depression patients’ life, depression is likewise associated with higher mortality and a shorter interval to recurrent stroke [4, 5]. The underlying molecular pathways involved in depressive disorders contain neuroinflammation hypothesis, monoamine neurotransmitter hypothesis, neurotrophic hypothesis, and hypothalamus-pituitary-adrenal axis (HPA) dysfunction hypothesis [6].

In the neuroinflammation hypothesis, high levels of inflammatory mediators, containing cytokines and chemokines and reactive oxygen species, are closely correlated with the development of PSD [7]. Many individuals with cerebrovascular ischemic events have been found to present a long-term and persistent pro-inflammatory background [8].

The immediate activation of central and peripheral immune inflammatory responses after acute stroke is followed by a significantly increased expression of pro-inflammatory cytokines, containing tumor necrosis factor (TNF)-α, interleukin (IL)-1, IL-6, etc. The increment of pro-inflammatory cytokines leads to the amplification of inflammatory response [9] and induces the overactivity of the HPA axis, as well as induces the noradrenergic dysfunction and widespread activation of indoleamine 2,3-dioxygenase. These actions exacerbate the depletion of 5-hydroxytryptamine (5-HT) in the temporal cortex and left frontal, which eventually leads to the depression [10]. Therefore, neuroinflammation following ischemic stroke plays important roles in the pathological process of PSD.

The present review focused on the relationship between neuroinflammation and PSD to reveal the duality of microglia and astrocytes in the pathological process of PSD and provide the potential therapeutic targets of PSD.

## 2. Post-stroke depression (PSD)

PSD is associated with sleep disorders, disability, cognitive impairment, poor rehabilitation outcomes, social withdrawal and isolation, and increased mortality [11]. The depression has been a focus since treatise “Depressive States in Old age”, which was reported by Gaupp et al. in 1905 [12]. The link between depression and atherosclerotic disease was firstly founded in 1955, the researcher described that atherosclerotic disease can lead to depressive disorders [13]. Subsequently, depression is found to be markedly more common in stroke patients [14], which attracts more attention of researches.

### 2.1 The definition and diagnosis of PSD

PSD is diagnosed and defined by the Diagnostic and Statistical Manual of Mental Disorders, Fifth Edition (DSM-5) criteria. A person must present at least five out of following nine symptoms: feelings of guilt, a sad mood, decreased energy levels, insomnia, decreased appetite, decreased concentration, changes in psychomotor activity (decreased or increased), and recurrent thoughts of self-harm or suicide and loss of interest in pleasurable activities (anhedonia) that lasts for at least 2 weeks [15].

Nevertheless, DSM-5 is not used for some exceptional cases when diagnosing patients do not exhibit typical psychological symptoms and cannot describe their psychological conditions coherently [16]. Therefore, it is often challenging for medical practitioners to diagnose such exceptional cases as PSD after a stroke. After decades of improvement and development, Hamilton Depression Rating Scale (HDRS) has been proved validity and reliability in screening for depressive symptoms among stroke patients [17]. In addition, Patient Health Questionnaire-9 (PHQ-9) has been justified and confirmed useful in measure depressive symptoms [18, 19].

### 2.2 The frequency of PSD

PSD occurs at any time point following stroke. A clinical study about 186 acute stroke patients in hospitals have reported that 31.72% of these patients were identified as suffering from depression within 2 weeks after stroke onset [15]. Mitchell et al. have reported in their comprehensive meta-analysis that one-third stroke patients had depressive disorders after a stroke [20]. 39% to 52% of stroke patients have at least one episode of depression within 5 years after stroke [5]. This variation is considered to be associated with differences in the diagnostic method of PSD, time point of depression assessment and study population [21].

### 2.3 The frequency of PSD and lesion location

The relationship between PSD and lesion characteristics has aroused considerable interest of researchers. Among the several hypotheses about the frequency of PSD and lesion location, one suggests that there are differences in the frequency of PSD between lesions in the right and left hemispheres [22]. Importantly, it has been suggested that stroke occurred in the left hemisphere cortex, particularly in frontal region, is related to higher risk for depression. In a clinical study, researchers have reported that female gender, left-sided stroke lesions and the absence of hypertension are statistically significant predictors of early onset of PSD [23]. Bekhbat et al. have confirmed that women with stroke are more likely to be diagnosed with PSD [24].

A clinical study has reported that higher incidence of depression tends to be associated with left hemisphere lesion and the severity of PSD is obviously correlated with the lesion to the frontal pole in the left anterior. Meanwhile, the frequency of depression in stroke patients with left posterior lesion is higher than that in patients with right anterior lesion [17]. Besides, Starkstein et al. have found that stroke patients with left subcortical or cortical anterior lesion have more serious depression and greater frequency than patients with lesion in other parts of the brain. Furthermore, a strong correlation between the lesion to the frontal pole and severity of depression was observed in both left cortical and subcortical lesions [25].

Accumulating studies have revealed that left hemisphere, particularly the left basal ganglia and frontal lobe, are key regions for PSD development [26, 27]. Klingbeil et al. have confirmed the significant association between PSD and the infarcts in the left ventrolateral prefrontal cortex [28]. A study involving 243 stroke patients have revealed that the severity of PSD is closely correlated with the extent of damage in the bilateral basal ganglia and left frontal lobe [29]. Thus, the incidence rate of PSD is strongly related to the location of lesion that triggers stroke.

Terroni and others have found the correlation between depression pathophysiology and dysfunction of the medial prefrontal cortex and of the left limbic-cortical-striatal-pallidal-thalamic (LCSPT) circuit in 2011 [30]. This hypothesis has been proposed by another study in 2021 that damage in LCSPT circuit predisposes to PSD [31]. The LCSPT circuits are of particular interest due to their vital role in the major depressive disorder (MDD) [32]. LCSPT circuit might likewise be disturbed indirectly by subsequent degeneration if it is outside the main ischemic lesion. Brain regions such as substantia nigra and the ipsilateral thalamus would exhibit delayed shrinkage on account of synaptic connections to the initial lesion location. For instance, anterograde degeneration results from basal ganglia lesions, retrograde degeneration results from cortical lesions [33].

Robinson et al. have found that PSD is related to the ischemic lesion of amine-containing axons, which is ascending from the brainstem to the left cerebral cortex and induces the decreased synthesis of norepinephrine (NE) and 5-HT in the limbic areas of frontal and temporal lobes and basal ganglia [17, 30, 34, 35]. Nevertheless, it must be pointed out that PSD is influenced by plenty of factors beyond stroke characteristics alone. The severity and development of PSD are related to the individual factors, containing pre-existing depression, post-stroke functional impairments, age, and disease duration[36-38].

## 3. The PSD and neuroinflammation

Accumulating evidence supports that inflammation is the dominant presence in depressive disorders [39]. Signs of systemic inflammation have been found in atypical depression, including higher circulating levels of IL-1β, TNF-α and C-reactive protein, as well as containing the dominance of IL-2 positive Th1 lymphocytes. In addition to the increased circulating proinflammatory cytokines, the typical systemic acute-phase reaction has likewise been described in the depression [40]. The pro-inflammatory factors perpetuate the nitrosative and oxidative stress in the central nervous system (CNS), which results in the consumption of glutathione, coenzyme Q10, n-3 polyunsaturated fatty acids (n-3 PUFA), and antioxidant capacity. These actions lead to significant activation of N-methyl-D-aspartate (NMDA) receptor and reduce 5-HT level [39].

Astrocytes and microglia in the CNS play vital roles in the pathological process of PSD via producing the cytokines, containing IL, TNF and interferon (IFN). Inflammation triggers depression via affecting the synthesis and secretion of monoamine neurotransmitters [41, 42]. Spalletta et al. explore and put forward the “cytokine hypothesis”, the investigators find that pro-inflammatory cytokines such as IL-1β, TNF-α and IL-18 interact with 5-HT, which results in the amplification of inflammatory processes in the limbic region and activates the indoleamine-2,3-dioxygenase (IDO) [43]. Activation of IDO in the limbic region promotes the conversion of tryptophan to kynurenine, which then leads to 5-HT depletion in the paralimbic structures. 5-HT depletion-induced physiological dysfunction is closely related to the occurrence of PSD [44].

### 3.1 Cytokines and PSD

#### 3.1.1 Pro-inflammatory cytokines and PSD (Fig. 1)

In an ischemic stroke, pro-inflammatory cytokines, containing IL-1β, IL-6, and TNF-α, are released from glial cells and/or neurons and trigger the inflammation [45-47]. Cerebral ischemia-induced inflammation directly exacerbate the pathologies via injuring the blood-brain barrier (BBB) and promoting neuronal cell death [45, 47].

Accumulating literatures have explored the relationship between the cytokines and PSD [48-52]. IL-18, a novel biomarker for PSD, is independently associated with depressive symptoms after stroke [48, 52, 53]. The inactive precursor protein of IL-18 is constitutively expressed in nearly all cells [54]. IL-18 is activated when it is cleaved by caspase-1 upon appropriate stimuli, and further secreted into extracellular space [54]. The activated IL-18 promotes the production of IFN-γ and the other pro-inflammatory cytokines via binding to the receptor of IL-18 (IL-18R). However, the pro-inflammatory activity of IL-18 can be antagonized by a constitutively secreted IL-18 binding protein (IL-18BP), which is intrinsic inhibitor of IL-18 [55].

IL-18 is expressed in ependymal cells, astrocytes, microglia, and a limited subpopulation of neurons in brain regions under physiological conditions [56, 57]. After cerebral ischemia, increased level of IL-18 mRNA has been found in rat brain tissues [58, 59]. The roles of IL-18 in PSD were well demonstrated by Wu et al. The researchers found that increased IL-18 induces the depression-like behaviors of mice via promoting the IL18 receptor/ sodium-potassium-chloride co-transporter 1 (NKCC1) signaling pathway [60]. The primary function of NKCC1 is maintaining intracellular Cl^-^ concentrations in cells via transporting the Na^+^, K^+^ and Cl^-^ ions into cells [61]. Dysfunction of NKCC1 is associated with various psychiatric disorders including depression [62]. Membrane NKCC1 in the CNS is new player in mediating depressive behaviors induced by repeated stress via reversing gamma-aminobutyric acid (GABA) inhibition and reducing Cl^-^ concentrations in cells [63]. Inhibiting IL-18 and its downstream NKCC1 is a potential strategy for the prevention and treatment of PSD [60].

IL-1β is linked to the P2X receptor family, activation of the P2X7-NLRP3-IL-1β pathway is connected with depressive-like behaviors [64], and inhibition of P2X7 is connected with antidepressant-like effects [65]. Enhanced expressions of IL-1β, IL-18 and the NLRP3 inflammasome were found in the ischemic hippocampus of PSD model rats [66]. In PSD, increased IL-6 levels in sera of patients persist up to one year post-stroke diagnosis [67]. Higher IL-6 levels in plasma of stroke patients prominently correlate with symptom severity of depression three months post-stroke, suggesting that IL-6 can be used as a potential PSD biomarker [68]. Increased IL-6 impairs synaptic neurotransmission, disrupts the HPA axis, and reduces the neurotrophic factors [69-71]. Furthermore, IL-6 enhances IDO activity, decreases central 5-HT availability and activates the kynurenine pathway [72].

**Figure 1.**
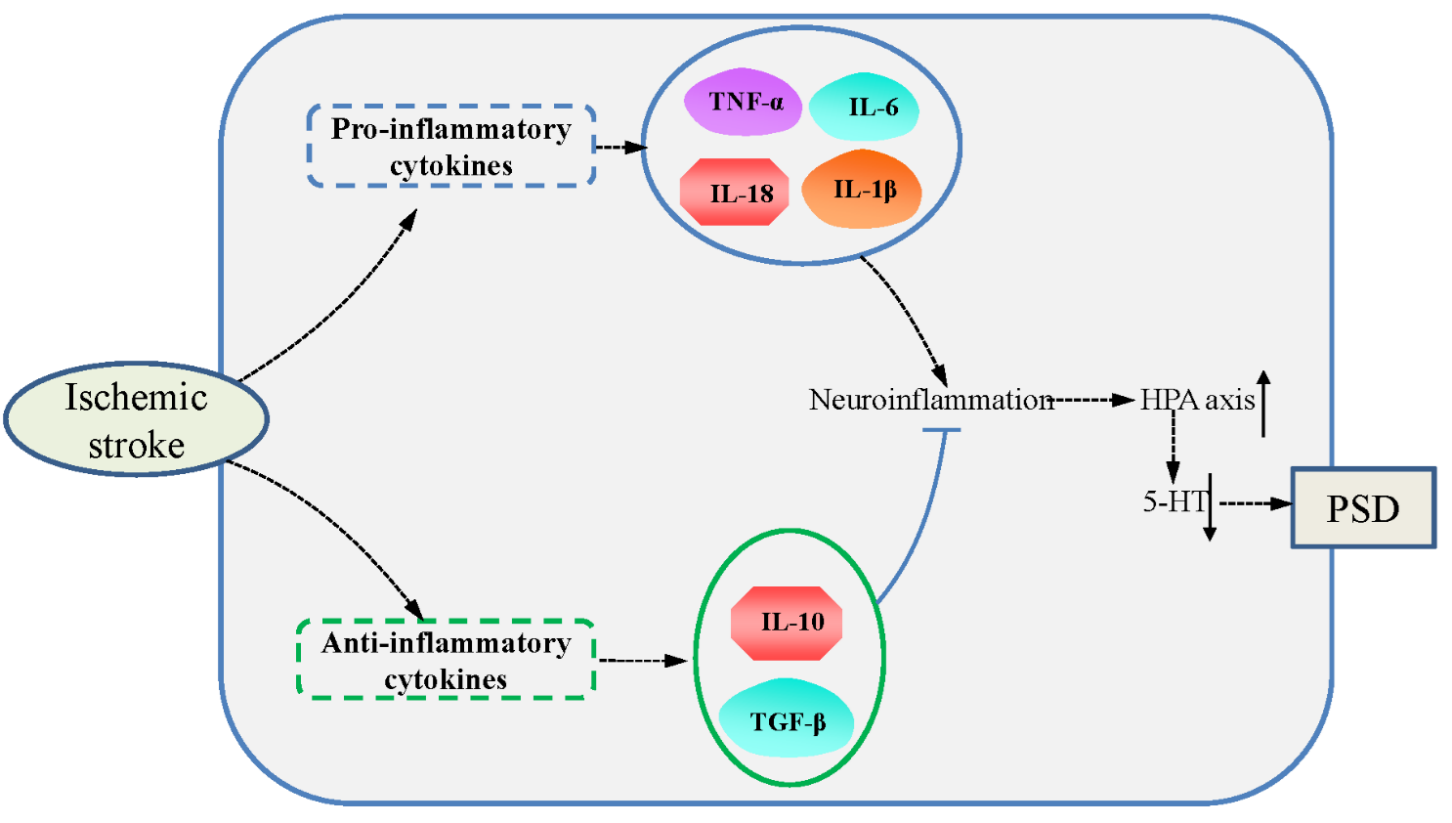
Cytokines and PSD.

#### 3.1.2 Anti-inflammatory cytokines and PSD (Fig. 1)

Decreased level of IL-10 is associated with depression and more severe somatic depressive symptoms [73]. Whereas, increased level of IL-10 has been reported in the MDD, potentially due to initial compensatory responses to acute inflammation [74]. IL-10 has inhibitory effects on the inflammation and can alleviate the depressive-like behavior in preclinical models [75, 76]. Patients with lower level of IL-10 have a greater possibility in developing PSD at one month after a stroke. Thus, IL-10 serves as an independent protective predictor for PSD [17]. The anti-inflammatory effects of IL-10 in the central nervous and peripheral system may be involved in the underlying mechanism of IL-10’s potential benefits in MDD [77].

Clinical studies have revealed that decreased IL-10 following stroke is associated with a decline in neurologic condition [78]. Chi et al. have found that IL-10 levels reduce markedly in patients with severe stroke compared to those with lighter stroke [79]. The researchers revealed a negative correlation between IL-10 levels and Hamilton rating scale for depression (HAMD) scores, and suggested that IL-10 can be used as a predictor for predicting PSD at a 1-month follow-up [79]. In addition, therapeutic potential of IL-10 is shown in patients with depression, inhibition of IL-10 signaling via administrating the neutralizing antibody against IL-10 led to prolonged depression-like behaviors in mice, suggesting that promoting IL-10 signaling may be a novel and efficacious therapeutic strategy to promote the resolution of depression [80].

Besides, another anti-inflammatory cytokine, trans-forming growth factor-β (TGF-β) is also connected with depression. Although there is controversy regarding TGF-β level in depression, researches have revealed that patients with MDD exhibit reduction of TGF-β network-associated gene transcripts in the choroid plexus [81] and reduced levels of TGF-β in serum [82, 83]. Importantly, the pro-inflammatory factors and the anti-inflammatory factors are antagonistic and bound up with the development direction of PSD [79, 84, 85]. For instance, Bensimon et al. reveal that pro-/anti-inflammatory ratios of IL-18/IL-10, IFN-γ/IL-10, and IL-1β/IL-10 are increased in sera of patients with moderately severe PSD [86]. The use of pro-/anti-inflammatory ratios (IL-1β/IL-10, TNF-α /IL-10, IL-18/IL-10, IL-6/IL-10, IFN-γ/IL-10) are also supported by Levada et al., they propose that the pro-/anti-inflammatory ratios might be used to differentiate PSD patients from non-PSD subjects [87]. Therefore, up-regulation of anti-inflammatory factors can antagonize the pro-inflammatory mediators on the PSD development and may be used to intervene PSD.

### 3.2 Microglia and PSD (Fig. 2)

As the resident immune cells in the CNS, microglia constantly monitor the surrounding microenvironment [88]. The ischemia stimuli after ischemic stroke induce the microglia activation, afterwards, microglia undergo physical and biochemical changes, including cell migration, phagocytosis, cell proliferation, and production of cytokines [89]. The dynamic alterations of microglia are associated with distinct functions and specific cytokines being secreted [90, 91]. Activated microglia have been defined as ‘M1 *vs* M2’phenotypes. M1 microglia exhibit proinflammatory effect and M2 microglia possess anti-inflammatory function [92]. Promoting the microglial polarization to the M2 phenotype exhibits the neuroprotective effects in the ischemic stroke [93-95]. Besides, polarization of M1/M2 microglia is closely associated with psychiatric disorders [96].

**Figure 2.**
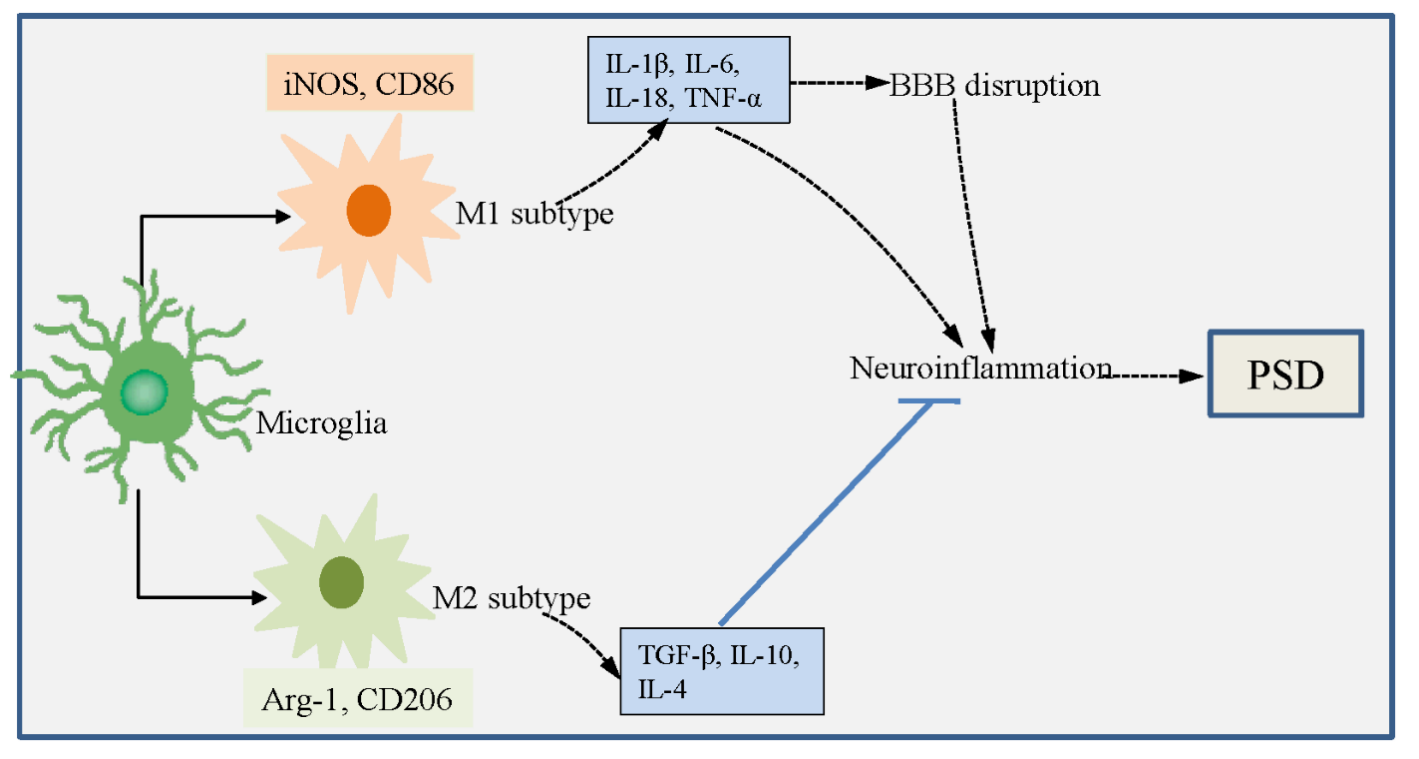
Dual roles of microglia in the PSD.

The main signal pathways that participating in the M1 microglial polarization: (1) helper T cells 1 (Th1)-secreted IFN-g promotes the transformation of microglia into M1 subtype via activating STAT and JAK1/JAK2 pathways [97]. (2) Activation of M1 microglia can be induced by another pathway triggered by lipopolysaccharide (LPS) or damage-associated molecular patterns (DAMPs) stimulation of TLR4. Subsequently, an “activation complex” is formed, which contains nuclear factor-κB (NF-κB), p65, p38, myeloid differentiation factor 88 (Myd88), and interferon regulatory factor 3 (IRF3). This complex in turn contributes to the polarization of M1 microglia [98]. The high number of TGF-β, IL-4, IL-10, regulatory T cells, and intracellular signaling via cAMP response element-binding protein (CREB) promote the differentiation of M2 microglia [99].

#### 3.2.1 M1 (pro-inflammatory) microglia and PSD

Microglia are polarized to M1 subtype within a short period of time after ischemic stroke, thereby secreting various pro-inflammatory factors, including IL-1β, IL-6, IL-18 and TNF-α. These pro-inflammatory cytokines recruit immune cells from blood to the damaged brain tissue, which can exacerbate the neuroinflammation and deteriorate the brain injury [100, 101]. Therefore, M1 microglia polarization promotes neuroinflammatory and immune reaction after ischemic stroke.

Inflammation after cerebral ischemia directly hinders the neural repair and exacerbates subsequent pathologies via deteriorating the BBB dysfunction and promoting neuronal cell death [45]. Among the cytokines related to PSD, IL-18 is mainly secreted by M1 microglia at a later phase and by neurons at early stage in mice model of depressive-like behaviors induced by LPS [102]. As aforementioned above, IL-18 is independently associated with depressive symptoms after stroke, and IL-18 level in sera of patients is a biomarker for PSD [48].

In addition to the vital roles of IL-18 on the PSD, cytokine hypothesis of PSD shows associations between PSD and the other microglia-produced TNF-α, IL-6, and IL-1β [85]. Serum levels of IL-6 were significantly higher in patients with PSD than in those without PSD [49]. Similarly, significant evaluation of IL-6,TNF-α, and IFN-γ levels has been found in PSD patients one year after stroke [67]. Kang et al. have reported that higher IL-6 and IL-18 levels in a sample of 286 participants were independently associated with depression within 2 weeks and at one year after stroke [48]. Inhibition of M1 microglial polarization can reduce the release of IL-1β, TNF-α and IL-6 [103]. Therefore, inhibiting the polarization of M1 microglia can reduce the occurrence of PSD.

#### 3.2.2 M2 microglia and PSD

The polarization of M2 microglia is characterized by the increased expressions of anti-inflammatory factors, containing TGF-β, IL-10 and IL-4 [104]. M2 microglia can be identified by detecting the expressions of arginase-1 (Arg-1) and CD206 [105]. In addition to the anti-inflammatory roles of IL-10 in the inflammatory response after stroke [106], IL-10 is also a negative regulator of pro-inflammatory cytokines TNF-α, IL-1β and IL-6 [107].

Zhang et al. have reported that intracerebroventricular administration of recombinant TGF-β improves the social isolation-exacerbated PSD and post-stroke anxiety (PSA), and promotes the hippocampal neurogenesis and cognitive functions [108]. Li et al. have revealed the enhanced expression of M1 microglia markers (iNOS, CD86, TNF-α and IL-1) in the rat hippocampal region in the PSD group than those in sham and poststroke groups. Besides, authors revealed that the M2 microglia marker Arg1 is prominently up-regulated in the PSD group than in the poststroke group [109]. Considering the distinct effects of M1 and M2 microglia in the depression, we proposed that inducing the phenotypic transformation of microglia to the M2 subtype is a potential therapeutic target for PSD.

### 3.3 PSD and astrocyte (Fig. 3)

Astrocytes are strongly associated with depression, and rapidly undergo morphological changes following ischemic stroke, such as hypertrophy and hyperplasia [110].

**Figure 3.**
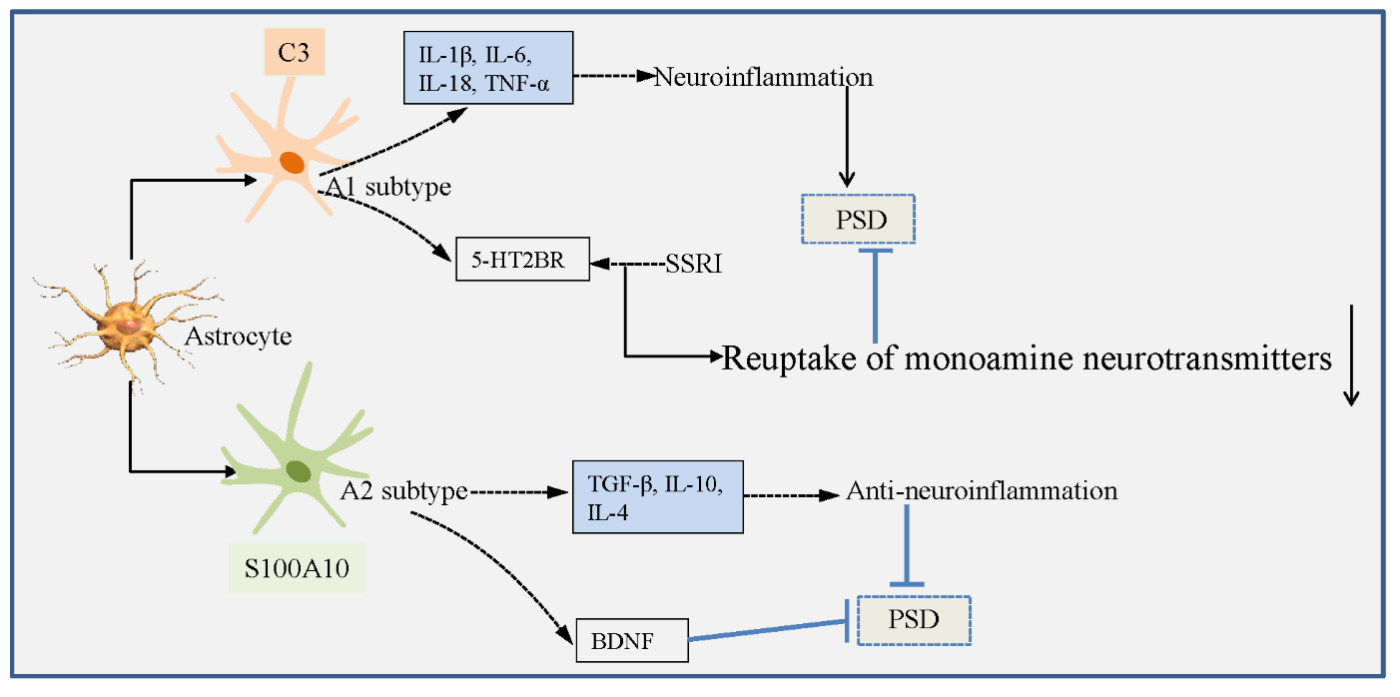
Dual roles of astrocytes in the PSD.

Ischemia stimuli and microglia-mediated inflammatory reaction trigger the activation of astrocytes, which then polarize into A1 subtype identified by C3 expression and A2 subtypes identified by S100A10 expression [111, 112]. A1 astrocytes-secreted IL-1β, IL-6, IL-18 and TNF-α aggravate the neuroinflammation and deteriorate the brain tissue damage [113, 114].Whereas, A2 astrocytes produce brain-derived neurotrophic factor (BDNF), which is known as neurotrophic factor and provide neuroprotection against cerebral ischemia injury [115-117]. BDNF is essential for the differentiation of neural stem cells (NSCs), and promote neuronal growth and maturation [118, 119].

Astrocyte markers would change in relation to emotion in brain regions of depressed patients. For instance, the decreased expression of glial fibrillary acidic protein (GFAP) is found in dorsolateral prefrontal cortex [120], ventrolateral prefrontal cortex (vPFC) [121], hippocampus [122], and prefrontal cortex [123] of depressed patients. In addition, there is increased level of S100β produced by A1 astrocytes [124] in serum [125], cerebrospinal fluid (CSF) [126], PFC [127] and hippocampus [128]. Importantly, the lower density of GFAP immunoreactive cells is found in the brain tissues of young patients compared with age-matched controls, this change does not exhibit in older patients with depression. Therefore, the changes of astrocyte markers are age dependent in depression patients [122, 129]. Effective treatment of depressive disorder could promote the proliferation of astrocytes [130, 131]. Whereas, dysfunction of astrocytes in medial prefrontal cortices leads to the depression like behaviors in rodents model [132, 133].

#### 3.3.1 Negative roles of astrocytes in PSD

The monoamine hypothesis of PSD suggests that underlying pathophysiologic basis of PSD occurrence is closely related to the reduced syntheses of monoamine neurotransmitter and correlated with conduction block in the basal ganglia, frontal, and temporal regions following ischemic brain injury. For example, 5-HT levels in serum and CSF of PSD patients are less than those in serum and CSF of stroke patients without depression [134, 135]. Single-nucleotide polymorphisms in genes of 5-HT transporter and 5-HT receptor have been found to be related to PSD [136]. Importantly, antidepressants such as selective 5-HT reuptake inhibitors (SSRIs), 5-HT and norepinephrine reuptake inhibitors (SNRIs) relieve the PSD symptoms via targeting the monoamine [137, 138].

Recent studies have revealed that A1 astrocytes participate in acute stress and LPS-induced depression-like behaviors. Besides, both 5-HT transporters and 5-HT receptors expressed in A1 astrocytes are targets of monoamine antidepressants [76, 139]. Other researches have revealed that 5-HT receptor modulators and SSRI mediate the activity of 5-HT_2B_ receptor (5-HT_2B_R) and downregulate the 5-HT receptor type-7 (5-HT7R) and 5-HT1A receptor (5-HT1AR) in astrocytes [140, 141], these actions reduce the expressions of connexin 43 (Cx43) on the plasma membrane of astroglia and decrease glutamate diffusion.

Fang et al. have revealed that inhibition of A1 astrocytes response participates in the antidepressant action of fluoxetine in a mouse model of MDD through astrocytic 5-HT2BR/β-arrestin2 signaling [142]. Thus, A1 astrocytes may be targets of antidepressants. Furthermore, A1 astrocytes-produced pro-inflammatory cytokines, containing IL-1β, IL-6, IL-18 and TNF-α [143], are likewise closely associated with the mechanisms of PSD [76, 144, 145].

#### 3.3.2 Positive roles of astrocytes in PSD

Glutamate excitotoxicity is found to be related to PSD development. Enhanced content of glutamate are found in the serum and PFC of PSD patients [146], which is similar to the result obtained from rodent models that increased level of glutamate and decreased level of GABA play a crucial role in the occurrence and development of PSD [147, 148]. Meanwhile, blood glutamate scavengers such as pyruvate and oxaloacetate can alleviate the depressive symptoms in the animal models of PSD [149]. Astrocytes participate in balancing the secretion and uptake of glutamate [150]. Excitatory amino acid transporter 2 (EAAT2) shared by astrocytes and neurons is a glutamate transporter and clear 90% of glutamate overflow in the synaptic cleft in the mammalian brain [151, 152]. In rodents, glial glutamate transporter-1 (GLT-1) is responsible for glutamate clearance. The expression of GLT-1 in astrocytes was significantly decreased in a rat model for PSD compared with control rats [151]. Therefore, astrocytes are involved in the PSD development via disrupting the glutamate circulation.

In addition, astrocytes likewise ameliorate the PSD by releasing BDNF [153]. The level of BDNF in serum from PSD patients at 3-6 months after stroke onset is lower than that from control stroke patients, suggesting that BDNF level in serum may be used as a predictor of PSD [154]. Moreover, the results of meta-analysis studies have shown that BDNF concentrations in patient sera significantly decrease during the early period of stroke and can predispose the patients to PSD [155, 156].

Further studies have confirmed that BDNF is released by A2 astrocytes but not by A1 astrocytes [157], and the reduced BDNF is related to the development of PSD, which is evidenced by the findings that PSD patients have reduced BDNF levels compared with stroke patients without depression [158, 159]. Besides, a reduced serum BDNF level has been found to enhance the PSD risk in the acute phase of stroke [160]. Moreover, the relationship between BDNF expression and antidepressants has been extensively explored. BDNF levels in sera of patients are increased after antidepressants treatment, containing SSRIs, monoamine oxidase inhibitors, tricyclic agents, serotonin-norepinephrine reuptake inhibitors, as well as including specific serotonergic antidepressants [161-163]. Importantly, injecting the exogenous BDNF into hippocampus can ameliorate the depression symptoms [164]. Not surprisingly, BDNF depletion can abolish the protective effects of antidepressants [161]. Inversely, overexpression of BDNF in hippocampus of rats can effectively alleviate the depression-like behaviors after stroke [165].

## 4. PSD and neurotransmitters

The neurotransmitter theory, a theory of the depression pathogenesis, posits the biological basis of PSD, which is related to the imbalance of the 5-HT, dopamine (DA), and NE systems [166]. During PSD, axons containing DA, NE and 5-HT between the brainstem and cerebral cortex might be impaired, leading to neurotransmitter production imbalances throughout the brain [34, 167]. Increased DA can lead to the dopaminergic activation, which promotes the synaptic plasticity and ameliorates the cognitive impairment [168]. DA neurons highly express neurotrophic factor Neuregulin 1 (NRG-1) receptors throughout development into adulthood. NRG-1 likewise affects the DA neurotransmission via raising extracellular DA levels [169].

The NE system in the CNS integrates inputs from lots of brain regions, it likewise projects widely to these brain regions [170]. The NE projections promotes the regrowth processes of 5-HT after neurotoxin treatment [171]. As aforementioned, SSRIs (selective 5-HT reuptake inhibitors) and SNRIs (5-HT) and NE reuptake inhibitors) relieve the PSD symptoms via targeting the 5-HT and NE [137, 138]. In a mice model of PSD after a stroke in the left medial prefrontal cortex (mPFC), the authors have found that Fluoxetine-mediated behavioral and cognitive recovery is associated with promoting the NE and 5-HT and NE axonal plasticity in these regions [172].

## 5. Conclusion

PSD impacts almost one-third of stroke survivors, impairing the recovery of patients. Neuroinflammation is one of the essential factors involved in the pathogenic mechanisms of ischemic stroke. Besides, neuroinflammation exerts an important role in the development of depression and is regarded as a sign of depression. Astrocytes are known as underlying therapeutic targets for depression and stroke due to their important roles in modulating neurotrophic factor expression, neurotransmission, and inflammation. In addition, microglia can monitor the CNS microenvironment and remove dead and dying neurons in time, which contributes to the supportive roles of microglia in the CNS homeostasis. Under the cerebral ischemia stimuli, both astrocytes and microglia undergo phenotypic transformation to different subtypes, A1/A2 and M1/M2, respectively. The different subtypes of microglia and astrocytes exert distinct (harmful or beneficial) roles in the PSD. Promoting the A2 astrocytes or M2 microglia polarization can offer opportunities for developing effective interventions for PSD.

## References

[1] GaeteJM, BogousslavskyJ (2008). Post-stroke depression. Expert Rev Neurother, 8:75-92.18088202 10.1586/14737175.8.1.75

[2] StaubF, BogousslavskyJ (2001). Post-stroke depression or fatigue. Eur Neurol, 45:3-5.11205620 10.1159/000052081

[3] PaolucciS, IosaM, CoiroP, VenturieroV, SavoA, De AngelisD, et al. (2019). Post-stroke Depression Increases Disability More Than 15% in Ischemic Stroke Survivors: A Case-Control Study. Front Neurol, 10:926.31507525 10.3389/fneur.2019.00926PMC6718567

[4] SiboltG, CurtzeS, MelkasS, PohjasvaaraT, KasteM, KarhunenPJ, et al. (2013). Post-stroke depression and depression-executive dysfunction syndrome are associated with recurrence of ischaemic stroke. Cerebrovasc Dis, 36:336-343.24193249 10.1159/000355145

[5] AyerbeL, AyisS, WolfeCD, RuddAG (2013). Natural history, predictors and outcomes of depression after stroke: systematic review and meta-analysis. Br J Psychiatry, 202:14-21.23284148 10.1192/bjp.bp.111.107664

[6] LoubinouxI, KronenbergG, EndresM, Schumann-BardP, FreretT, FilipkowskiRK, et al. (2012). Post-stroke depression: mechanisms, translation and therapy. J Cell Mol Med, 16:1961-1969.22348642 10.1111/j.1582-4934.2012.01555.xPMC3822966

[7] FanQ, LiuY, ShengL, LvS, YangL, ZhangZ, et al. (2023). Chaihu-Shugan-San inhibits neuroinflammation in the treatment of post-stroke depression through the JAK/STAT3-GSK3beta/PTEN/Akt pathway. Biomed Pharmacother, 160:114385.36774722 10.1016/j.biopha.2023.114385

[8] KliperE, BashatDB, BornsteinNM, Shenhar-TsarfatyS, HalleviH, AurielE, et al. (2013). Cognitive decline after stroke: relation to inflammatory biomarkers and hippocampal volume. Stroke, 44:1433-1435.23444307 10.1161/STROKEAHA.111.000536

[9] EmsleyHC, SmithCJ, GavinCM, GeorgiouRF, VailA, BarberanEM, et al. (2003). An early and sustained peripheral inflammatory response in acute ischaemic stroke: relationships with infection and atherosclerosis. J Neuroimmunol, 139:93-101.12799026 10.1016/s0165-5728(03)00134-6

[10] ChenH, LiuF, SunD, ZhangJ, LuoS, LiaoQ, et al. (2022). The potential risk factors of early-onset post-stroke depression from immuno-inflammatory perspective. Front Immunol, 13:1000631.36225923 10.3389/fimmu.2022.1000631PMC9549963

[11] HadidiN, Treat-JacobsonDJ, LindquistR (2009). Poststroke depression and functional outcome: a critical review of literature. Heart Lung, 38:151-162.19254633 10.1016/j.hrtlng.2008.05.002

[12] GauppR, BerriosGE, Pomarol-ClotetE (2000). Depressive states in old age. (Classic Text No. 42). Hist Psychiatry, 11:213-225.11678103 10.1177/0957154X0001104205

[13] RothM (1955). The natural history of mental disorder in old age. J Ment Sci, 101:281-301.13243044 10.1192/bjp.101.423.281

[14] FolsteinMF, MaibergerR, McHughPR (1977). Mood disorder as a specific complication of stroke. J Neurol Neurosurg Psychiatry, 40:1018-1020.591971 10.1136/jnnp.40.10.1018PMC492887

[15] ChenH, HuangX, ZengC, SunD, LiuF, ZhangJ, et al. (2023). The role of indoleamine 2,3-dioxygenase 1 in early-onset post-stroke depression. Front Immunol, 14:1125634.36911716 10.3389/fimmu.2023.1125634PMC9998486

[16] TrivediMH (2004). The link between depression and physical symptoms. Prim Care Companion J Clin Psychiatry, 6:12-16.16001092 PMC486942

[17] RobinsonRG, KubosKL, StarrLB, RaoK, PriceTR (1984). Mood disorders in stroke patients. Importance of location of lesion. Brain, 107(Pt 1):81-93.6697163 10.1093/brain/107.1.81

[18] KroenkeK, SpitzerRL, WilliamsJB (2001). The PHQ-9: validity of a brief depression severity measure. J Gen Intern Med, 16:606-613.11556941 10.1046/j.1525-1497.2001.016009606.xPMC1495268

[19] SpitzerRL, KroenkeK, WilliamsJB (1999). Validation and utility of a self-report version of PRIME-MD: the PHQ primary care study. Primary Care Evaluation of Mental Disorders. Patient Health Questionnaire. JAMA, 282:1737-1744.10568646 10.1001/jama.282.18.1737

[20] MitchellAJ, ShethB, GillJ, YadegarfarM, StubbsB, YadegarfarM, et al. (2017). Prevalence and predictors of post-stroke mood disorders: A meta-analysis and meta-regression of depression, anxiety and adjustment disorder. Gen Hosp Psychiatry, 47:48-60.28807138 10.1016/j.genhosppsych.2017.04.001

[21] MedeirosGC, RoyD, KontosN, BeachSR (2020). Post-stroke depression: A 2020 updated review. Gen Hosp Psychiatry, 66:70-80.32717644 10.1016/j.genhosppsych.2020.06.011

[22] SinyorD, AmatoP, KaloupekDG, BeckerR, GoldenbergM, CoopersmithH (1986). Post-stroke depression: relationships to functional impairment, coping strategies, and rehabilitation outcome. Stroke, 17:1102-1107.3810708 10.1161/01.str.17.6.1102

[23] BaccaroA, WangYP, BrunoniAR, CandidoM, ConfortoAB, da Costa LeiteC, et al. (2019). Does stroke laterality predict major depression and cognitive impairment after stroke? Two-year prospective evaluation in the EMMA study. Prog Neuropsychopharmacol Biol Psychiatry, 94:109639.31075348 10.1016/j.pnpbp.2019.109639

[24] BekhbatM, NeighGN (2018). Sex differences in the neuro-immune consequences of stress: Focus on depression and anxiety. Brain Behav Immun, 67:1-12.28216088 10.1016/j.bbi.2017.02.006PMC5559342

[25] StarksteinSE, RobinsonRG, PriceTR (1987). Comparison of cortical and subcortical lesions in the production of poststroke mood disorders. Brain, 110(Pt 4):1045-1059.3651794 10.1093/brain/110.4.1045

[26] KimJS, Choi-KwonS (2000). Poststroke depression and emotional incontinence: correlation with lesion location. Neurology, 54:1805-1810.10802788 10.1212/wnl.54.9.1805

[27] MorrisPL, RobinsonRG, RaphaelB, HopwoodMJ (1996). Lesion location and poststroke depression. J Neuropsychiatry Clin Neurosci, 8:399-403.9116475 10.1176/jnp.8.4.399

[28] KlingbeilJ, BrandtML, WawrzyniakM, StockertA, SchneiderHR, BaumP, et al. (2021). Association of Lesion Location and Depressive Symptoms Poststroke. Stroke, 52:830-837.33504189 10.1161/STROKEAHA.120.031889

[29] HamaS, YamashitaH, ShigenobuM, WatanabeA, KurisuK, YamawakiS, et al. (2007). Post-stroke affective or apathetic depression and lesion location: left frontal lobe and bilateral basal ganglia. Eur Arch Psychiatry Clin Neurosci, 257:149-152.17131217 10.1007/s00406-006-0698-7

[30] TerroniL, AmaroE, IosifescuDV, TinoneG, SatoJR, LeiteCC, et al. (2011). Stroke lesion in cortical neural circuits and post-stroke incidence of major depressive episode: a 4-month prospective study. World J Biol Psychiatry, 12:539-548.21486107 10.3109/15622975.2011.562242PMC3279135

[31] LiangW, FanZ, CuiS, ShenX, WangL (2021). The association between White matter microstructure alterations detected by Diffusional kurtosis imaging in Neural circuit and post-stroke depression. Neurol Res, 43:535-542.33588692 10.1080/01616412.2021.1888033

[32] LuY, LiangH, HanD, MoY, LiZ, ChengY, et al. (2016). The volumetric and shape changes of the putamen and thalamus in first episode, untreated major depressive disorder. Neuroimage Clin, 11:658-666.27222797 10.1016/j.nicl.2016.04.008PMC4873692

[33] DihneM, GrommesC, LutzenburgM, WitteOW, BlockF (2002). Different mechanisms of secondary neuronal damage in thalamic nuclei after focal cerebral ischemia in rats. Stroke, 33:3006-3011.12468804 10.1161/01.str.0000039406.64644.cb

[34] SantosM, KovariE, GoldG, BozikasVP, HofPR, BourasC, et al. (2009). The neuroanatomical model of post-stroke depression: towards a change of focus? J Neurol Sci, 283:158-162.19264329 10.1016/j.jns.2009.02.334PMC2915758

[35] NarushimaK, KosierJT, RobinsonRG (2003). A reappraisal of poststroke depression, intra- and inter-hemispheric lesion location using meta-analysis. J Neuropsychiatry Clin Neurosci, 15:422-430.14627768 10.1176/jnp.15.4.422

[36] GuoJ, WangJ, SunW, LiuX (2022). The advances of post-stroke depression: 2021 update. J Neurol, 269:1236-1249.34052887 10.1007/s00415-021-10597-4

[37] IlutS, StanA, BlesneagA, VacarasV, VesaS, FodoreanuL (2017). Factors that influence the severity of post-stroke depression. J Med Life, 10:167-171.29075345 PMC5652262

[38] KaraahmetOZ, GurcayE, AvlukOC, UmayEK, GundogduI, EcerkaleO, et al. (2017). Poststroke depression: risk factors and potential effects on functional recovery. Int J Rehabil Res, 40:71-75.28099186 10.1097/MRR.0000000000000210

[39] LeonardB, MaesM (2012). Mechanistic explanations how cell-mediated immune activation, inflammation and oxidative and nitrosative stress pathways and their sequels and concomitants play a role in the pathophysiology of unipolar depression. Neurosci Biobehav Rev, 36:764-785.22197082 10.1016/j.neubiorev.2011.12.005

[40] MaesM (1993). A review on the acute phase response in major depression. Rev Neurosci, 4:407-416.7506108 10.1515/revneuro.1993.4.4.407

[41] OglodekE (2022). Changes in the Serum Levels of Cytokines: IL-1beta, IL-4, IL-8 and IL-10 in Depression with and without Posttraumatic Stress Disorder. Brain Sci, 12.35326343 10.3390/brainsci12030387PMC8946076

[42] WangL, WangR, LiuL, QiaoD, BaldwinDS, HouR (2019). Effects of SSRIs on peripheral inflammatory markers in patients with major depressive disorder: A systematic review and meta-analysis. Brain Behav Immun, 79:24-38.30797959 10.1016/j.bbi.2019.02.021

[43] SpallettaG, BossuP, CiaramellaA, BriaP, CaltagironeC, RobinsonRG (2006). The etiology of poststroke depression: a review of the literature and a new hypothesis involving inflammatory cytokines. Mol Psychiatry, 11:984-991.16894392 10.1038/sj.mp.4001879

[44] LiR, FanW, LiD, LiuX (2022). Correlation of common inflammatory cytokines with cognition impairment, anxiety, and depression in acute ischemic stroke patients. Braz J Med Biol Res, 55:e11517.35239774 10.1590/1414-431X2021e11517PMC8905669

[45] ChamorroA, DirnaglU, UrraX, PlanasAM (2016). Neuroprotection in acute stroke: targeting excitotoxicity, oxidative and nitrosative stress, and inflammation. Lancet Neurol, 15:869-881.27180033 10.1016/S1474-4422(16)00114-9

[46] IadecolaC, AnratherJ (2011). The immunology of stroke: from mechanisms to translation. Nat Med, 17:796-808.21738161 10.1038/nm.2399PMC3137275

[47] ShichitaT, ItoM, YoshimuraA (2014). Post-ischemic inflammation regulates neural damage and protection. Front Cell Neurosci, 8:319.25352781 10.3389/fncel.2014.00319PMC4196547

[48] KangHJ, BaeKY, KimSW, KimJT, ParkMS, ChoKH, et al. (2016). Effects of interleukin-6, interleukin-18, and statin use, evaluated at acute stroke, on post-stroke depression during 1-year follow-up. Psychoneuroendocrinology, 72:156-160.27428088 10.1016/j.psyneuen.2016.07.001

[49] SpallettaG, CravelloL, ImperialeF, SalaniF, BossuP, PicchettoL, et al. (2013). Neuropsychiatric symptoms and interleukin-6 serum levels in acute stroke. J Neuropsychiatry Clin Neurosci, 25:255-263.24247852 10.1176/appi.neuropsych.12120399

[50] HannestadJ, DellaGioiaN, BlochM (2011). The effect of antidepressant medication treatment on serum levels of inflammatory cytokines: a meta-analysis. Neuropsychopharmacology, 36:2452-2459.21796103 10.1038/npp.2011.132PMC3194072

[51] KimJM, StewartR, KimSW, ShinIS, KimJT, ParkMS, et al. (2012). Associations of cytokine gene polymorphisms with post-stroke depression. World J Biol Psychiatry, 13:579-587.21793642 10.3109/15622975.2011.588247

[52] YangL, ZhangZ, SunD, XuZ, ZhangX, LiL (2010). The serum interleukin-18 is a potential marker for development of post-stroke depression. Neurol Res, 32:340-346.20482998 10.1179/016164110X12656393665080

[53] BossuP, SalaniF, CacciariC, PicchettoL, CaoM, BizzoniF, et al. (2009). Disease outcome, alexithymia and depression are differently associated with serum IL-18 levels in acute stroke. Curr Neurovasc Res, 6:163-170.19534720 10.2174/156720209788970036

[54] DinarelloCA, NovickD, KimS, KaplanskiG (2013). Interleukin-18 and IL-18 binding protein. Front Immunol, 4:289.24115947 10.3389/fimmu.2013.00289PMC3792554

[55] NovickD, KimSH, FantuzziG, ReznikovLL, DinarelloCA, RubinsteinM (1999). Interleukin-18 binding protein: a novel modulator of the Th1 cytokine response. Immunity, 10:127-136.10023777 10.1016/s1074-7613(00)80013-8

[56] Kuwahara-OtaniS, MaedaS, KobayashiK, MinatoY, TanakaK, YamanishiK, et al. (2017). Interleukin-18 and its receptor are expressed in gonadotropin-releasing hormone neurons of mouse and rat forebrain. Neurosci Lett, 650:33-37.28373090 10.1016/j.neulet.2017.03.051

[57] SugamaS, ChoBP, BakerH, JohTH, LuceroJ, ContiB (2002). Neurons of the superior nucleus of the medial habenula and ependymal cells express IL-18 in rat CNS. Brain Res, 958:1-9.12468024 10.1016/s0006-8993(02)03363-2

[58] HedtjarnM, LeverinAL, ErikssonK, BlomgrenK, MallardC, HagbergH (2002). Interleukin-18 involvement in hypoxic-ischemic brain injury. J Neurosci, 22:5910-5919.12122053 10.1523/JNEUROSCI.22-14-05910.2002PMC6757918

[59] JanderS, SchroeterM, StollG (2002). Interleukin-18 expression after focal ischemia of the rat brain: association with the late-stage inflammatory response. J Cereb Blood Flow Metab, 22:62-70.11807395 10.1097/00004647-200201000-00008

[60] WuD, ZhangG, ZhaoC, YangY, MiaoZ, XuX (2020). Interleukin-18 from neurons and microglia mediates depressive behaviors in mice with post-stroke depression. Brain Behav Immun, 88:411-420.32272223 10.1016/j.bbi.2020.04.004

[61] RussellJM (2000). Sodium-potassium-chloride cotransport. Physiol Rev, 80:211-276.10617769 10.1152/physrev.2000.80.1.211

[62] GoubertE, AltvaterM, RoviraMN, KhalilovI, MazzarinoM, SebastianiA, et al. (2019). Bumetanide Prevents Brain Trauma-Induced Depressive-Like Behavior. Front Mol Neurosci, 12:12.30804751 10.3389/fnmol.2019.00012PMC6370740

[63] TsukaharaT, MasuharaM, IwaiH, SonomuraT, SatoT (2015). Repeated stress-induced expression pattern alterations of the hippocampal chloride transporters KCC2 and NKCC1 associated with behavioral abnormalities in female mice. Biochem Biophys Res Commun, 465:145-151.26239662 10.1016/j.bbrc.2015.07.153

[64] YueN, HuangH, ZhuX, HanQ, WangY, LiB, et al. (2017). Activation of P2X7 receptor and NLRP3 inflammasome assembly in hippocampal glial cells mediates chronic stress-induced depressive-like behaviors. J Neuroinflammation, 14:102.28486969 10.1186/s12974-017-0865-yPMC5424302

[65] ClarkAK, StanilandAA, MarchandF, KaanTK, McMahonSB, MalcangioM (2010). P2X7-dependent release of interleukin-1beta and nociception in the spinal cord following lipopolysaccharide. J Neurosci, 30:573-582.20071520 10.1523/JNEUROSCI.3295-09.2010PMC2880485

[66] LiZ, XuH, XuY, LuG, PengQ, ChenJ, et al. (2021). Morinda officinalis oligosaccharides alleviate depressive-like behaviors in post-stroke rats via suppressing NLRP3 inflammasome to inhibit hippocampal inflammation. CNS Neurosci Ther, 27:1570-1586.34559953 10.1111/cns.13732PMC8611777

[67] SuJA, ChouSY, TsaiCS, HungTH (2012). Cytokine changes in the pathophysiology of poststroke depression. Gen Hosp Psychiatry, 34:35-39.22055333 10.1016/j.genhosppsych.2011.09.020

[68] KorostynskiM, HoinkisD, PiechotaM, GoldaS, PeraJ, SlowikA, et al. (2021). Toll-like receptor 4-mediated cytokine synthesis and post-stroke depressive symptoms. Transl Psychiatry, 11:246.33903586 10.1038/s41398-021-01359-xPMC8076201

[69] TingEY, YangAC, TsaiSJ (2020). Role of Interleukin-6 in Depressive Disorder. Int J Mol Sci, 21.32235786 10.3390/ijms21062194PMC7139933

[70] JeonSW, KimYK (2016). Neuroinflammation and cytokine abnormality in major depression: Cause or consequence in that illness? World J Psychiatry, 6:283-293.27679767 10.5498/wjp.v6.i3.283PMC5031928

[71] GirottiM, DoneganJJ, MorilakDA (2013). Influence of hypothalamic IL-6/gp130 receptor signaling on the HPA axis response to chronic stress. Psychoneuroendocrinology, 38:1158-1169.23218517 10.1016/j.psyneuen.2012.11.004PMC3609893

[72] AndersonG, KuberaM, DudaW, LasonW, BerkM, MaesM (2013). Increased IL-6 trans-signaling in depression: focus on the tryptophan catabolite pathway, melatonin and neuroprogression. Pharmacol Rep, 65:1647-1654.24553013 10.1016/s1734-1140(13)71526-3

[73] EuteneuerF, DannehlK, Del ReyA, EnglerH, SchedlowskiM, RiefW (2017). Peripheral Immune Alterations in Major Depression: The Role of Subtypes and Pathogenetic Characteristics. Front Psychiatry, 8:250.29218020 10.3389/fpsyt.2017.00250PMC5703704

[74] WienerCD, MoreiraFP, PortelaLV, StrogulskiNR, LaraDR, da SilvaRA, et al. (2019). Interleukin-6 and Interleukin-10 in mood disorders: A population-based study. Psychiatry Res, 273:685-689.31207853 10.1016/j.psychres.2019.01.100

[75] LangheinM, Seitz-HollandJ, LyallAE, PasternakO, ChungaN, Cetin-KarayumakS, et al. (2022). Association between peripheral inflammation and free-water imaging in Major Depressive Disorder before and after ketamine treatment - A pilot study. J Affect Disord, 314:78-85.35779673 10.1016/j.jad.2022.06.043PMC11186306

[76] ZhangHY, WangY, HeY, WangT, HuangXH, ZhaoCM, et al. (2020). A1 astrocytes contribute to murine depression-like behavior and cognitive dysfunction, which can be alleviated by IL-10 or fluorocitrate treatment. J Neuroinflammation, 17:200.32611425 10.1186/s12974-020-01871-9PMC7331266

[77] NordenDM, FennAM, DuganA, GodboutJP (2014). TGFbeta produced by IL-10 redirected astrocytes attenuates microglial activation. Glia, 62:881-895.24616125 10.1002/glia.22647PMC4061706

[78] ProttiGG, GagliardiRJ, ForteWC, SprovieriSR (2013). Interleukin-10 may protect against progressing injury during the acute phase of ischemic stroke. Arq Neuropsiquiatr, 71:846-851.24394869 10.1590/0004-282X20130168

[79] ChiCH, HuangYY, YeSZ, ShaoMM, JiangMX, YangMY, et al. (2021). Interleukin-10 level is associated with post-stroke depression in acute ischaemic stroke patients. J Affect Disord, 293:254-260.34217963 10.1016/j.jad.2021.06.037

[80] LaumetG, EdralinJD, ChiangAC, DantzerR, HeijnenCJ, KavelaarsA (2018). Resolution of inflammation-induced depression requires T lymphocytes and endogenous brain interleukin-10 signaling. Neuropsychopharmacology, 43:2597-2605.30054585 10.1038/s41386-018-0154-1PMC6224384

[81] TurnerCA, ThompsonRC, BunneyWE, SchatzbergAF, BarchasJD, MyersRM, et al. (2014). Altered choroid plexus gene expression in major depressive disorder. Front Hum Neurosci, 8:238.24795602 10.3389/fnhum.2014.00238PMC4001046

[82] MusilR, SchwarzMJ, RiedelM, DehningS, CeroveckiA, SpellmannI, et al. (2011). Elevated macrophage migration inhibitory factor and decreased transforming growth factor-beta levels in major depression--no influence of celecoxib treatment. J Affect Disord, 134:217-225.21684012 10.1016/j.jad.2011.05.047

[83] SutcigilL, OktenliC, MusabakU, BozkurtA, CanseverA, UzunO, et al. (2007). Pro- and anti-inflammatory cytokine balance in major depression: effect of sertraline therapy. Clin Dev Immunol, 2007:76396.18317531 10.1155/2007/76396PMC2248234

[84] HuJ, ZhouW, ZhouZ, YangQ, HanJ, YanY, et al. (2019). [Predictive value of inflammatory indicators for post-stroke depression in patients with ischemic stroke]. Nan Fang Yi Ke Da Xue Xue Bao, 39:665-671.31270044 10.12122/j.issn.1673-4254.2019.06.06PMC6743917

[85] KimJM, KangHJ, KimJW, BaeKY, KimSW, KimJT, et al. (2017). Associations of Tumor Necrosis Factor-alpha and Interleukin-1beta Levels and Polymorphisms with Post-Stroke Depression. Am J Geriatr Psychiatry, 25:1300-1308.28844626 10.1016/j.jagp.2017.07.012

[86] BensimonK, HerrmannN, SwardfagerW, YiH, BlackSE, GaoFQ, et al. (2014). Kynurenine and depressive symptoms in a poststroke population. Neuropsychiatr Dis Treat, 10:1827-1835.25285006 10.2147/NDT.S65740PMC4181733

[87] LevadaOA, TroyanAS (2018). Poststroke Depression Biomarkers: A Narrative Review. Front Neurol, 9:577.30061860 10.3389/fneur.2018.00577PMC6055004

[88] SaijoK, GlassCK (2011). Microglial cell origin and phenotypes in health and disease. Nat Rev Immunol, 11:775-787.22025055 10.1038/nri3086

[89] HammondTR, DufortC, Dissing-OlesenL, GieraS, YoungA, WysokerA, et al. (2019). Single-Cell RNA Sequencing of Microglia throughout the Mouse Lifespan and in the Injured Brain Reveals Complex Cell-State Changes. Immunity, 50:253-271 e256.30471926 10.1016/j.immuni.2018.11.004PMC6655561

[90] WallerR, BaxterL, FillinghamDJ, CoelhoS, PozoJM, MozumderM, et al. (2019). Iba-1-/CD68+ microglia are a prominent feature of age-associated deep subcortical white matter lesions. PLoS One, 14:e0210888.30682074 10.1371/journal.pone.0210888PMC6347230

[91] BocheD, PerryVH, NicollJA (2013). Review: activation patterns of microglia and their identification in the human brain. Neuropathol Appl Neurobiol, 39:3-18.23252647 10.1111/nan.12011

[92] WangJ, XingH, WanL, JiangX, WangC, WuY (2018). Treatment targets for M2 microglia polarization in ischemic stroke. Biomed Pharmacother, 105:518-525.29883947 10.1016/j.biopha.2018.05.143

[93] HeC, LiuR, FanZ, LiY, YangM, WugangH, et al. (2021). Microglia in the Pathophysiology of Hemorrhagic Stroke and the Relationship Between Microglia and Pain After Stroke: A Narrative Review. Pain Ther, 10:927-939.34278548 10.1007/s40122-021-00288-3PMC8586130

[94] LiQ, DaiZ, CaoY, WangL (2019). Caspase-1 inhibition mediates neuroprotection in experimental stroke by polarizing M2 microglia/macrophage and suppressing NF-kappaB activation. Biochem Biophys Res Commun, 513:479-485.30979498 10.1016/j.bbrc.2019.03.202

[95] YangL, TuckerD, DongY, WuC, LuY, LiY, et al. (2018). Photobiomodulation therapy promotes neurogenesis by improving post-stroke local microenvironment and stimulating neuroprogenitor cells. Exp Neurol, 299:86-96.29056360 10.1016/j.expneurol.2017.10.013PMC5723531

[96] NakagawaY, ChibaK (2014). Role of microglial m1/m2 polarization in relapse and remission of psychiatric disorders and diseases. Pharmaceuticals (Basel), 7:1028-1048.25429645 10.3390/ph7121028PMC4276905

[97] RoeschS, RappC, DettlingS, Herold-MendeC (2018). When Immune Cells Turn Bad-Tumor-Associated Microglia/Macrophages in Glioma. Int J Mol Sci, 19.29389898 10.3390/ijms19020436PMC5855658

[98] ChuF, ShiM, ZhengC, ShenD, ZhuJ, ZhengX, et al. (2018). The roles of macrophages and microglia in multiple sclerosis and experimental autoimmune encephalomyelitis. J Neuroimmunol, 318:1-7.29606295 10.1016/j.jneuroim.2018.02.015

[99] Dos SantosIRC, DiasMNC, Gomes-LealW (2021). Microglial activation and adult neurogenesis after brain stroke. Neural Regen Res, 16:456-459.32985465 10.4103/1673-5374.291383PMC7996005

[100] ChenAQ, FangZ, ChenXL, YangS, ZhouYF, MaoL, et al. (2019). Microglia-derived TNF-alpha mediates endothelial necroptosis aggravating blood brain-barrier disruption after ischemic stroke. Cell Death Dis, 10:487.31221990 10.1038/s41419-019-1716-9PMC6586814

[101] SharmaM, ArbabzadaN, FloodPM (2019). Mechanism underlying beta2-AR agonist-mediated phenotypic conversion of LPS-activated microglial cells. J Neuroimmunol, 332:37-48.30933849 10.1016/j.jneuroim.2019.03.017

[102] ZhangL, PrevinR, LuL, LiaoRF, JinY, WangRK (2018). Crocin, a natural product attenuates lipopolysaccharide-induced anxiety and depressive-like behaviors through suppressing NF-kB and NLRP3 signaling pathway. Brain Res Bull, 142:352-359.30179677 10.1016/j.brainresbull.2018.08.021

[103] ChenT, LiZ, LiS, ZouY, GaoX, ShuS, et al. (2022). Cycloastragenol suppresses M1 and promotes M2 polarization in LPS-stimulated BV-2 cells and ischemic stroke mice. Int Immunopharmacol, 113:109290.36252498 10.1016/j.intimp.2022.109290

[104] OrihuelaR, McPhersonCA, HarryGJ (2016). Microglial M1/M2 polarization and metabolic states. Br J Pharmacol, 173:649-665.25800044 10.1111/bph.13139PMC4742299

[105] Hsu CH, Pan YJ, Zheng YT, Lo RY, Yang FY (2023). Ultrasound reduces inflammation by modulating M1/M2 polarization of microglia through STAT1/STAT6/PPARgamma signaling pathways. CNS Neurosci Ther. 10.1111/cns.14333 PMC10651950 37401041

[106] GarciaJM, StillingsSA, LeclercJL, PhillipsH, EdwardsNJ, RobicsekSA, et al. (2017). Role of Interleukin-10 in Acute Brain Injuries. Front Neurol, 8:244.28659854 10.3389/fneur.2017.00244PMC5466968

[107] XiaCY, ZhangS, GaoY, WangZZ, ChenNH (2015). Selective modulation of microglia polarization to M2 phenotype for stroke treatment. Int Immunopharmacol, 25:377-382.25704852 10.1016/j.intimp.2015.02.019

[108] ZhangY, YuP, LiuH, YaoH, YaoS, YuanSY, et al. (2019). Hyperforin improves post-stroke social isolation-induced exaggeration of PSD and PSA via TGF-beta. Int J Mol Med, 43:413-425.30387813 10.3892/ijmm.2018.3971PMC6257831

[109] WeiL, GuoJ, YuX, ChenH, DuY, JiZ, et al. (2021). Role and characteristics of hippocampal region microglial activation in poststroke depression. J Affect Disord, 291:270-278.34058609 10.1016/j.jad.2021.05.022

[110] LiddelowSA, GuttenplanKA, ClarkeLE, BennettFC, BohlenCJ, SchirmerL, et al. (2017). Neurotoxic reactive astrocytes are induced by activated microglia. Nature, 541:481-487.28099414 10.1038/nature21029PMC5404890

[111] ScarisbrickIA, RadulovicM, BurdaJE, LarsonN, BlaberSI, GianniniC, et al. (2012). Kallikrein 6 is a novel molecular trigger of reactive astrogliosis. Biol Chem, 393:355-367.22505518 10.1515/hsz-2011-0241PMC3335747

[112] HerxLM, YongVW (2001). Interleukin-1 beta is required for the early evolution of reactive astrogliosis following CNS lesion. J Neuropathol Exp Neurol, 60:961-971.11589427 10.1093/jnen/60.10.961

[113] PonathG, RamananS, MubarakM, HousleyW, LeeS, SahinkayaFR, et al. (2017). Myelin phagocytosis by astrocytes after myelin damage promotes lesion pathology. Brain, 140:399-413.28007993 10.1093/brain/aww298PMC5841057

[114] LianH, YangL, ColeA, SunL, ChiangAC, FowlerSW, et al. (2015). NFkappaB-activated astroglial release of complement C3 compromises neuronal morphology and function associated with Alzheimer's disease. Neuron, 85:101-115.25533482 10.1016/j.neuron.2014.11.018PMC4289109

[115] YunSP, KamTI, PanickerN, KimS, OhY, ParkJS, et al. (2018). Block of A1 astrocyte conversion by microglia is neuroprotective in models of Parkinson's disease. Nat Med, 24:931-938.29892066 10.1038/s41591-018-0051-5PMC6039259

[116] MorizawaYM, HirayamaY, OhnoN, ShibataS, ShigetomiE, SuiY, et al. (2017). Reactive astrocytes function as phagocytes after brain ischemia via ABCA1-mediated pathway. Nat Commun, 8:28.28642575 10.1038/s41467-017-00037-1PMC5481424

[117] SekarA, BialasAR, de RiveraH, DavisA, HammondTR, KamitakiN, et al. (2016). Schizophrenia risk from complex variation of complement component 4. Nature, 530:177-183.26814963 10.1038/nature16549PMC4752392

[118] ParkH, PooMM (2013). Neurotrophin regulation of neural circuit development and function. Nat Rev Neurosci, 14:7-23.23254191 10.1038/nrn3379

[119] LazarovO, MattsonMP, PetersonDA, PimplikarSW, van PraagH (2010). When neurogenesis encounters aging and disease. Trends Neurosci, 33:569-579.20961627 10.1016/j.tins.2010.09.003PMC2981641

[120] O'LearyLA, BelliveauC, DavoliMA, MaJC, TantiA, TureckiG, et al. (2021). Widespread Decrease of Cerebral Vimentin-Immunoreactive Astrocytes in Depressed Suicides. Front Psychiatry, 12:640963.33613346 10.3389/fpsyt.2021.640963PMC7890082

[121] RajkowskaG, LegutkoB, MoulanaM, SyedM, RomeroDG, StockmeierCA, et al. (2018). Astrocyte pathology in the ventral prefrontal white matter in depression. J Psychiatr Res, 102:150-158.29660602 10.1016/j.jpsychires.2018.04.005PMC6005746

[122] CobbJA, O'NeillK, MilnerJ, MahajanGJ, LawrenceTJ, MayWL, et al. (2016). Density of GFAP-immunoreactive astrocytes is decreased in left hippocampi in major depressive disorder. Neuroscience, 316:209-220.26742791 10.1016/j.neuroscience.2015.12.044PMC4836620

[123] NagyC, SudermanM, YangJ, SzyfM, MechawarN, ErnstC, et al. (2015). Astrocytic abnormalities and global DNA methylation patterns in depression and suicide. Mol Psychiatry, 20:320-328.24662927 10.1038/mp.2014.21PMC5293540

[124] HuangC, ZhangF, LiP, SongC (2022). Low-Dose IL-2 Attenuated Depression-like Behaviors and Pathological Changes through Restoring the Balances between IL-6 and TGF-beta and between Th17 and Treg in a Chronic Stress-Induced Mouse Model of Depression. Int J Mol Sci, 23.36430328 10.3390/ijms232213856PMC9699071

[125] TuralU, IrvinMK, IosifescuDV (2022). Correlation between S100B and severity of depression in MDD: A meta-analysis. World J Biol Psychiatry, 23:456-463.34854356 10.1080/15622975.2021.2013042

[126] HideseS, HattoriK, SasayamaD, TsumagariT, MiyakawaT, MatsumuraR, et al. (2020). Cerebrospinal fluid neuroplasticity-associated protein levels in patients with psychiatric disorders: a multiplex immunoassay study. Transl Psychiatry, 10:161.32439851 10.1038/s41398-020-0843-5PMC7242469

[127] ZhangL, VerwerRWH, ZhaoJ, HuitingaI, LucassenPJ, SwaabDF (2021). Changes in glial gene expression in the prefrontal cortex in relation to major depressive disorder, suicide and psychotic features. J Affect Disord, 295:893-903.34706460 10.1016/j.jad.2021.08.098

[128] GosT, SchroeterML, LesselW, BernsteinHG, DobrowolnyH, SchiltzK, et al. (2013). S100B-immunopositive astrocytes and oligodendrocytes in the hippocampus are differentially afflicted in unipolar and bipolar depression: a postmortem study. J Psychiatr Res, 47:1694-1699.23896207 10.1016/j.jpsychires.2013.07.005

[129] CuiY, YangY, NiZ, DongY, CaiG, FoncelleA, et al. (2018). Astroglial Kir4.1 in the lateral habenula drives neuronal bursts in depression. Nature, 554:323-327.29446379 10.1038/nature25752

[130] ZhaoYF, VerkhratskyA, TangY, IllesP (2022). Astrocytes and major depression: The purinergic avenue. Neuropharmacology, 220:109252.36122663 10.1016/j.neuropharm.2022.109252

[131] PengL, VerkhratskyA, GuL, LiB (2015). Targeting astrocytes in major depression. Expert Rev Neurother, 15:1299-1306.26471936 10.1586/14737175.2015.1095094

[132] YaoS, XuMD, WangY, ZhaoST, WangJ, ChenGF, et al. (2023). Astrocytic lactate dehydrogenase A regulates neuronal excitability and depressive-like behaviors through lactate homeostasis in mice. Nat Commun, 14:729.36759610 10.1038/s41467-023-36209-5PMC9911790

[133] LiuJ, MoJW, WangX, AnZ, ZhangS, ZhangCY, et al. (2022). Astrocyte dysfunction drives abnormal resting-state functional connectivity in depression. Sci Adv, 8:eabo2098.36383661 10.1126/sciadv.abo2098PMC9668300

[134] ZhangX, WangCB, DuanLH, LongJJ, XiaoP, WangYL, et al. (2023). Correlation research of serum substance P, CCK-8, and 5-HT values with depression levels in stroke survivors. Eur Rev Med Pharmacol Sci, 27:1248-1254.36876663 10.26355/eurrev_202302_31357

[135] GaoHQ, ZhuHY, ZhangYQ, WangLX (2008). Reduction of cerebrospinal fluid and plasma serotonin in patients with post-stroke depression: A preliminary report. Clin Invest Med, 31:E351-356.19032905 10.25011/cim.v31i6.4921

[136] ZhaoQ, GuoY, YangD, YangT, MengX (2016). Serotonin Transporter Gene 5-HTTLPR Polymorphism as a Protective Factor Against the Progression of Post-Stroke Depression. Mol Neurobiol, 53:1699-1705.25700622 10.1007/s12035-015-9120-7

[137] LeggLA, RudbergAS, HuaX, WuS, HackettML, TilneyR, et al. (2021). Selective serotonin reuptake inhibitors (SSRIs) for stroke recovery. Cochrane Database Syst Rev, 11:CD009286.34780067 10.1002/14651858.CD009286.pub4PMC8592088

[138] VillaRF, FerrariF, MorettiA (2018). Post-stroke depression: Mechanisms and pharmacological treatment. Pharmacol Ther, 184:131-144.29128343 10.1016/j.pharmthera.2017.11.005

[139] ZhangJ, NingL, WangJ (2020). Dietary quercetin attenuates depressive-like behaviors by inhibiting astrocyte reactivation in response to stress. Biochem Biophys Res Commun, 533:1338-1346.33059918 10.1016/j.bbrc.2020.10.016

[140] ShiroyamaT, FukuyamaK, OkadaM (2021). Distinct Effects of Escitalopram and Vortioxetine on Astroglial L-Glutamate Release Associated with Connexin43. Int J Mol Sci, 22.34576176 10.3390/ijms221810013PMC8468507

[141] ChenB, ZhangM, JiM, GongW, ChenB, ZorecR, et al. (2021). The Association Between Antidepressant Effect of SSRIs and Astrocytes: Conceptual Overview and Meta-analysis of the Literature. Neurochem Res, 46:2731-2745.33527219 10.1007/s11064-020-03225-6

[142] FangY, DingX, ZhangY, CaiL, GeY, MaK, et al. (2022). Fluoxetine inhibited the activation of A1 reactive astrocyte in a mouse model of major depressive disorder through astrocytic 5-HT(2B)R/beta-arrestin2 pathway. J Neuroinflammation, 19:23.35093099 10.1186/s12974-022-02389-yPMC8800238

[143] FangY, GuoH, WangQ, LiuC, GeS, YanB (2022). The role and mechanism of NLRP3 inflammasome-mediated astrocyte activation in dehydrocorydaline against CUMS-induced depression. Front Pharmacol, 13:1008249.36506556 10.3389/fphar.2022.1008249PMC9726715

[144] BauerME, TeixeiraAL (2019). Inflammation in psychiatric disorders: what comes first? Ann N Y Acad Sci, 1437:57-67.29752710 10.1111/nyas.13712

[145] ShariqAS, BrietzkeE, RosenblatJD, BarendraV, PanZ, McIntyreRS (2018). Targeting cytokines in reduction of depressive symptoms: A comprehensive review. Prog Neuropsychopharmacol Biol Psychiatry, 83:86-91.29309829 10.1016/j.pnpbp.2018.01.003

[146] ChengSY, ZhaoYD, LiJ, ChenXY, WangRD, ZengJW (2014). Plasma levels of glutamate during stroke is associated with development of post-stroke depression. Psychoneuroendocrinology, 47:126-135.25001962 10.1016/j.psyneuen.2014.05.006

[147] ZhaoA, MaB, XuL, YaoM, ZhangY, XueB, et al. (2021). Jiedu Tongluo Granules Ameliorates Post-stroke Depression Rat Model via Regulating NMDAR/BDNF Signaling Pathway. Front Pharmacol, 12:662003.34093193 10.3389/fphar.2021.662003PMC8173625

[148] FrankD, KutsR, TsenterP, GruenbaumBF, GrinshpunY, ZvenigorodskyV, et al. (2019). The effect of pyruvate on the development and progression of post-stroke depression: A new therapeutic approach. Neuropharmacology, 155:173-184.31153808 10.1016/j.neuropharm.2019.05.035PMC6863053

[149] GruenbaumBF, KutzR, ZlotnikA, BoykoM (2020). Blood glutamate scavenging as a novel glutamate-based therapeutic approach for post-stroke depression. Ther Adv Psychopharmacol, 10:2045125320903951.32110376 10.1177/2045125320903951PMC7026819

[150] RappeneauV, BlakerA, PetroJR, YamamotoBK, ShimamotoA (2016). Disruption of the Glutamate-Glutamine Cycle Involving Astrocytes in an Animal Model of Depression for Males and Females. Front Behav Neurosci, 10:231.28018190 10.3389/fnbeh.2016.00231PMC5147055

[151] YuD, ChengZ, AliAI, WangJ, LeK, ChibaatarE, et al. (2019). Down-expressed GLT-1 in PSD astrocytes inhibits synaptic formation of NSC-derived neurons in vitro. Cell Cycle, 18:105-114.30558468 10.1080/15384101.2018.1560201PMC6343715

[152] Martinez-LozadaZ, GuillemAM, RobinsonMB (2016). Transcriptional Regulation of Glutamate Transporters: From Extracellular Signals to Transcription Factors. Adv Pharmacol, 76:103-145.27288076 10.1016/bs.apha.2016.01.004PMC5544923

[153] JiangMQ, YuSP, WeiZZ, ZhongW, CaoW, GuX, et al. (2021). Conversion of Reactive Astrocytes to Induced Neurons Enhances Neuronal Repair and Functional Recovery After Ischemic Stroke. Front Aging Neurosci, 13:612856.33841125 10.3389/fnagi.2021.612856PMC8032905

[154] LiJ, ZhaoYD, ZengJW, ChenXY, WangRD, ChengSY (2014). Serum Brain-derived neurotrophic factor levels in post-stroke depression. J Affect Disord, 168:373-379.25106034 10.1016/j.jad.2014.07.011

[155] XuHB, XuYH, HeY, XueF, WeiJ, ZhangH, et al. (2018). Decreased Serum Brain-Derived Neurotrophic Factor May Indicate the Development of Poststroke Depression in Patients with Acute Ischemic Stroke: A Meta-Analysis. J Stroke Cerebrovasc Dis, 27:709-715.29128330 10.1016/j.jstrokecerebrovasdis.2017.10.003

[156] NoonanK, CareyLM, CrewtherSG (2013). Meta-analyses indicate associations between neuroendocrine activation, deactivation in neurotrophic and neuroimaging markers in depression after stroke. J Stroke Cerebrovasc Dis, 22:e124-135.23149149 10.1016/j.jstrokecerebrovasdis.2012.09.008

[157] ZhaZ, LiuYJ, LiuSS, ZhangN, LiJL, QiF, et al. (2022). Bu Shen Yi Sui Capsule Promotes Myelin Repair by Modulating the Transformation of A1/A2 Reactive Astrocytes In Vivo and In Vitro. Oxid Med Cell Longev, 2022:3800004.36092158 10.1155/2022/3800004PMC9458373

[158] ZhangC, WangX, ZhuQ, MeiY, ZhangZ, XuH (2022). Decreased Serum Brain-Derived Neurotrophic Factor in Poststroke Depression: A Systematic Review and Meta-Analysis. Front Psychiatry, 13:876557.35664480 10.3389/fpsyt.2022.876557PMC9160429

[159] ShanD, ZhengY, FroudK (2021). Brain-Derived Neurotrophic Factor as a Clinical Biomarker in Predicting the Development of Post-Stroke Depression: A Review of Evidence. Cureus, 13:e15662.34141514 10.7759/cureus.15662PMC8204918

[160] LiangJ, YueY, JiangH, GengD, WangJ, LuJ, et al. (2018). Genetic variations in the p11/tPA/BDNF pathway are associated with post stroke depression. J Affect Disord, 226:313-325.29028593 10.1016/j.jad.2017.09.055

[161] BjorkholmC, MonteggiaLM (2016). BDNF - a key transducer of antidepressant effects. Neuropharmacology, 102:72-79.26519901 10.1016/j.neuropharm.2015.10.034PMC4763983

[162] Lee BH, Kim YK (2010). The roles of BDNF in the pathophysiology of major depression and in antidepressant treatment. Psychiatry Investig, 7:231-235. 10.4306/pi.2010.7.4.231 PMC3022308 21253405

[163] MartinowichK, LuB (2008). Interaction between BDNF and serotonin: role in mood disorders. Neuropsychopharmacology, 33:73-83.17882234 10.1038/sj.npp.1301571

[164] ShirayamaY, ChenAC, NakagawaS, RussellDS, DumanRS (2002). Brain-derived neurotrophic factor produces antidepressant effects in behavioral models of depression. J Neurosci, 22:3251-3261.11943826 10.1523/JNEUROSCI.22-08-03251.2002PMC6757539

[165] ChenHH, ZhangN, LiWY, FangMR, ZhangH, FangYS, et al. (2015). Overexpression of brain-derived neurotrophic factor in the hippocampus protects against post-stroke depression. Neural Regen Res, 10:1427-1432.26604903 10.4103/1673-5374.165510PMC4625508

[166] ShahZA, SharmaP, VohoraSB (2003). Ginkgo biloba normalises stress-elevated alterations in brain catecholamines, serotonin and plasma corticosterone levels. Eur Neuropsychopharmacol, 13:321-325.12957329 10.1016/s0924-977x(03)00005-1

[167] KumarU, Medel-MatusJS, RedwineHM, ShinD, HenslerJG, SankarR, et al. (2016). Effects of selective serotonin and norepinephrine reuptake inhibitors on depressive- and impulsive-like behaviors and on monoamine transmission in experimental temporal lobe epilepsy. Epilepsia, 57:506-515.26813337 10.1111/epi.13321PMC4783206

[168] LavergneF, JayTM (2010). A new strategy for antidepressant prescription. Front Neurosci, 4:192.21151361 10.3389/fnins.2010.00192PMC2995552

[169] SkirzewskiM, KaravanovaI, ShamirA, ErbenL, Garcia-OlivaresJ, ShinJH, et al. (2018). ErbB4 signaling in dopaminergic axonal projections increases extracellular dopamine levels and regulates spatial/working memory behaviors. Mol Psychiatry, 23:2227-2237.28727685 10.1038/mp.2017.132PMC5775946

[170] SchwarzLA, MiyamichiK, GaoXJ, BeierKT, WeissbourdB, DeLoachKE, et al. (2015). Viral-genetic tracing of the input-output organization of a central noradrenaline circuit. Nature, 524:88-92.26131933 10.1038/nature14600PMC4587569

[171] LiuY, IshidaY, ShinodaK, NakamuraS (2003). Interaction between serotonergic and noradrenergic axons during axonal regeneration. Exp Neurol, 184:169-178.14637090 10.1016/s0014-4886(03)00221-8

[172] ZahraiA, Vahid-AnsariF, DaigleM, AlbertPR (2020). Fluoxetine-induced recovery of serotonin and norepinephrine projections in a mouse model of post-stroke depression. Transl Psychiatry, 10:334.32999279 10.1038/s41398-020-01008-9PMC7527452

